# Modulation of granulocyte-endothelium interactions by antileukoproteinase: inhibition of anti-type II collagen antibody-induced leukocyte attachment to the synovial endothelium

**DOI:** 10.1186/ar1973

**Published:** 2006-06-15

**Authors:** Bettina Sehnert, Philip Gierer, Saleh Ibrahim, Anja Kühl, Reinhard Voll, Kutty Selva Nandakumar, Rikard Holmdahl, Rupert Hallmann, Brigitte Vollmar, Harald Burkhardt

**Affiliations:** 1 Department of Internal Medicine III and Institute of Clinical Immunology at the Friedrich-Alexander-University of Erlangen-Nürnberg, Krankenhausstrasse 12, 91054 Erlangen, Germany; 2 Department of Experimental Surgery, University of Rostock, Schillingallee 70, 18055 Rostock, Germany; 3 Department of Trauma and Reconstructive Surgery, University of Rostock, Schillingallee 70, 18055 Rostock, Germany; 4 Institute of Immunology, University of Rostock, Schillingallee 70, 18055 Rostock, Germany; 5 IZKF Research Group N2, Nikolaus-Fiebiger Center, and Department of Internal Medicine III, Friedrich-Alexander-University of Erlangen-Nürnberg, Glückstrasse 6, 91054 Erlangen, Germany; 6 Section for Medical Inflammation Research, BMC I11, Lund University, Sweden; 7 Institute for Physiological Chemistry and Pathobiochemistry, University of Münster, Waldeyerstrasse 15, 48149 Münster, Germany; 8 Current address: Division of Rheumatology, Johann Wolfgang Goethe University Frankfurt, Theodor-Stern-Kai 7, 60590 Frankfurt am Main, Germany

## Abstract

Antileukoproteinase (ALP) is a physiological inhibitor of granulocytic serine proteases that has been shown to have anti-inflammatory properties in addition to its antiproteolytic activity. On the basis of its potential to block anti-collagen type II (CII) antibody-induced arthritis (CAIA) and to suppress the conformational activation of β_2_-integrins in leukocytes, the present study was undertaken to investigate its interference with leukocyte adherence to cytokine-activated endothelium. The potential of recombinant ALP to block the interactions of leukocytes with the endothelial lining was concomitantly investigated *in vitro *and *in vivo*. Thus, intravital fluorescence microscopic imaging of leukocyte rolling and firm adhesion to postcapillary venules were performed in the knee joints of DBA1/J mice after intravenous injection of anti-CII mAbs. An IL-1β-activated endothelial layer formed by a murine glomerular cell line (glEND.2) was used to assay the interaction with human leukocytes *in vitro*. Electromobility shift and luciferase reporter gene assays permitted the analysis of cytokine-induced activation of the NF-κB pathway. Fluorescence-activated cell sorting was applied to determine endothelial E-selectin expression. Leukocyte rolling and firm adhesion to the synovial endothelium in an early response to the anti-CII antibody transfer were significantly decreased in ALP-pretreated mice. Concomitantly, ALP suppressed the IL-1β-induced NF-κB activation and the upregulation of E-selectin expression in glEND.2 cells *in vitro*. These findings support the notion that the newly uncovered properties of ALP to interfere with cytokine signalling and upregulation of adhesion molecules in endothelial cells are likely to contribute to the therapeutic potential of ALP in immune-complex-induced tissue injury.

## Introduction

Antileukoproteinase (ALP), also named secretory leukocyte protease inhibitor (SLPI), is a highly basic (pI > 10), acid-stable inhibitor of neutrophil serine proteinases (molecular mass 11 kDa) [1]. Originally described as a specific protector against proteolytic attack of the upper respiratory and urogenital tract, the physiological expression of ALP has also been demonstrated for a variety of extramucosal cells, including neutrophils [2] and macrophages [3]. In addition to antiproteolytic properties, ALP exerts a variety of anti-inflammatory actions on monocytes and neutrophils [4,5]. It has also been proposed that the ability of ALP to downregulate the lipopolysaccharide response in macrophages is most probably related to an inhibitory effect on NF-κB activation [6]. More recently it has been demonstrated that treatment with ALP significantly decreased the incidence and severity of anti-collagen type II (CII) antibody-induced arthritis (CAIA). In addition to clinical amelioration, decreased leukocytic infiltration of synovial tissue and protective effects on cartilage and bone erosion in ALP-treated mice was seen [5]. The effect on leukocyte extravasation and tissue destruction could be ascribed, at least partly, to the interference of ALP with cytoskeletal changes in Fc-receptor-stimulated granulocytes [5]. The downmodulation of stimulation-induced F-actin assembly in neutrophils through the interaction of ALP with the actin-bundling protein l-plastin caused a blockade of the conformational activation of β_2_-integrins [5].

Because regulating the avidity of leukocyte β_2_-integrins LFA-1 (CD11a/CD18) and Mac-1 (CD11b/CD18) is critical for the recruitment of leukocytes into inflamed tissues [7-9] we focused the present investigation of ALP effects on the initial steps of leukocyte interaction with the endothelial layer after an inflammatory stimulus. A statistical analysis of intravital microscopic images recorded from the knee joints of untreated control mice during an early time span of 24 hours after the transfer of anti-CII mAbs [10] revealed an antibody-induced increase in leukocyte adhesion to the vessel walls. Preventive administration of ALP as a single dose of 100 μg per mouse led to a significant suppression of leukocyte rolling on, and of firm adhesion to, the synovial venular endothelium. Accompanying experiments *in vitro *with IL-1β-activated endothelial cells demonstrated the capacity of ALP to suppress the cytokine-induced increase in leukocyte adhesion. Moreover, an inhibitory effect of ALP preincubation on IL-1β-induced endothelial E-selectin surface expression was recorded by fluorescence-activated cell sorting (FACS) analysis of anti-E-selectin antibody-stained glEND.2 cells (a murine glomerular cell line). The subsequent elucidation of a suppressive effect of ALP on IL-1β-induced NF-κB activation in glEND.2 cells also suggests that the modulation of this signalling pathway is probably involved in the ALP-dependent inhibition of IL-1β-induced E-selectin expression. Moreover, the observed suppression of leukocyte rolling *in vivo *as a selectin-dependent interaction with the vessel wall [11] is easily reconcilable with the newly uncovered blocking effect of ALP on a crucial cytokine signalling pathway in endothelial cells [12]. This regulatory potential and the inhibition of firm leukocyte adhesion *in vivo *by ALP, which probably reflects the already known interference with β_2_-integrin activation on leukocytes, complement each other in their anti-inflammatory effect. This synergism may contribute to the established capacity of ALP to block leukocyte infiltration and tissue injury in a variety of experimental models of inflammation, such as streptococcal cell wall arthritis [13], CAIA [5] or ischaemia/reperfusion-induced organ damage [14].

## Materials and methods

### Anti-CII mAb transfer

The experimental protocol was approved by the local animal ethics committee and followed the National Institutes of Health guidelines for the care and use of laboratory animals. DBA1/J mice were obtained from the Jackson Laboratory (Bar Harbor, ME, USA). All mice were kept under standard conditions at the animal care facility of the University of Rostock.

For the induction of immune-complex-induced inflammation, 14-week-old mice (*n *= 8 per group) received two mAbs (CIIC1 and M2.139 [5,15]; 4.5 mg of each) intravenously into the caudal vein. Simultaneously the animals were treated with either 100 or 300 μg of recombinant human ALP [5,16] (kindly provided by Dr Heinzel-Wieland, Darmstadt, Germany) or 300 μg of a control protein (human serum albumin; Octapharma, Langenfeld, Germany), or received an equal volume of saline only. After 6 and 24 hours, animals (*n *= 6 to 8 per time point) were anaesthetized with ketamine (90 mg/kg body weight) and xylacin (6 mg/kg) for subsequent *in vivo *multifluorescence microscopy. Animals were placed on a heating pad to maintain a body temperature of 37°C and a catheter was placed in the left jugular vein for the application of fluorescent dyes. For *in vivo *multifluorescence microscopy of synovial microcirculation, the knee joint model was used as described [17]. In brief, skin was incised distal to the patella tendon. After removal of the overlying soft tissues, the patella tendon was cut transversely and the proximal and distal parts carefully mobilized. After exposure, the Hoffa's fatty body was superfused with physiological saline solution ay 37°C to prevent tissues from drying and was finally covered with a glass slide. After a 15-minute stabilization period after surgical preparation, *in vivo *microscopy of the synovial tissue was performed. At the end of each experiment the animals were killed by exsanguination.

### Recombinant ALP

Human ALP was produced as a recombinant protein in *Escherichia coli *and subsequently purified to homogeneity by a multistep purification protocol by ion-exchange, metal-chelate and size-exclusion chromatography as originally described by Heinzel-Wieland and colleagues [16]. Analytical reverse-phase chromatography revealed a single peak, and SDS-PAGE exhibited a single band with the expected electrophoretic mobility on being stained with silver. The material was tested for inhibition of granulocyte elastase and cathepsin G. To ensure the removal of any endotoxin contamination, the ALP charges were run on detoxi-gel columns (polymyxin B-conjugated columns; Perbio Science, Bonn, Germany) in accordance with the manufacturer's instructions. Routine testing of the final ALP preparations before use demonstrated that for all analysed samples the endotoxin content remained below the detection limit (0.05 U/ml) of the applied *Limulus *amoebocyte lysate gel-clot assay (Sigma, Taufkirchen, Germany).

### *In vivo *fluorescence microscopy

After intravenous injection of fluorescein isothiocyanate (FITC)-labelled dextran (15 mg/kg body weight) (Sigma, Deisenhofen, Germany) and rhodamine 6G (0.15 mg/kg body weight) (Sigma), *in vivo *microscopy was performed with a Zeiss microscope (Axiotech Vario 100HD; Carl Zeiss, Jena, Germany) equipped with a 100 W mercury lamp and filter sets for blue (excitation at 465 to 495 nm; emission at more than 505 nm) and green (excitation at 510 to 560 nm; emission at more than 575 nm) epi-illumination. By the use of water-immersion objectives (× 20 W/numerical aperture (NA) 0.5 and × 40 W/NA 0.8; Zeiss) final magnifications of × 306 and × 630 were achieved. Images were recorded by means of a charge-coupled device video camera (FK 6990-IQ-S; Pieper, Schwerte, Germany) and transferred to an S-VHS video system for subsequent off-line analysis.

### Microcirculatory analysis

For quantitative off-line analysis a computer-assisted microcirculation image analysis system was used (CapImage v.7.4; Zeintl, Heidelberg, Germany). For the assessment of interaction between leukocytes and endothelial cells in postcapillary venules, the flow behavior of leukocytes was analysed with respect to free floating, rolling and adherent leukocytes [17,18]. Rolling leukocytes were defined as those cells moving along the vessel wall at a velocity less than 40% of that of leukocytes at the centreline and were expressed as a percentage of the total leukocyte flux. Venular leukocyte adherence was defined as the number of leukocytes not moving or detaching from the endothelial lining of the venular vessel wall during an observation period of 20 seconds. Assuming cylindrical microvessel geometry, leukocyte adherence was expressed as non-moving cells per endothelial surface (*n*/mm^2^), calculated from the diameter and length of the vessel segment analysed.

### Statistical analysis

Results are given as means ± SEM. After proving the assumption of normality, comparisons between the experimental groups were performed by one-way analysis of variance (ANOVA), followed by the appropriate post-hoc multiple comparison procedure, including Bonferroni correction. Statistical significance was set at *p *< 0.05.

### Isolation of human granulocytes

Blood cells were obtained from heparinized venous blood from healthy donors. In brief, granulocytes were isolated by discontinuous Percoll density gradient centrifugation with 70% and 62% Percoll (Biochrom, Berlin, Germany). The phase containing the granulocytes was separately transferred to a new tube and washed with PBS. Contaminating erythrocytes were removed by hypotonic lysis and a subsequent centrifugation step. The purified granulocytes were recovered from the cell pellet and resuspended in DMEM at a final concentration of 10^6 ^cells/ml.

### Leukocytes adhesion assay

The leukocyte adhesion assays were performed as described by Hammel and colleagues [19], with slight modifications. glEND.2 cells were produced and characterized as described for mlEND.1 cells [20]. They were seeded on Labtek chamber slides (6 × 10^4^) and grown to confluence. glEND.2 cells were incubated for 2 hours with different concentrations of ALP (100 nM to 5 μM). The upregulation of adhesion molecules on the glEND.2 cells was induced by activating the cells with IL-1β (20 ng/ml, R&D Systems GmbH, Wiesbaden, Germany) for 4 hours at 37°C. The cells were washed intensively with DMEM and the freshly isolated human granulocytes were added in a volume of 200 μl to the endothelial cell layer and agitated horizontally at 75 r.p.m. for 20 minutes at 4°C. Cells were washed carefully three times in DMEM and fixed overnight in 2.5% glutardialdehyde (Roth, Karlsruhe, Germany). On the next day, the adherent cells were evaluated by direct microscopic counting of four randomly chosen visual fields. Some experiments were performed with the human endothelial cell line EA.hy 926 that was originally derived by the fusion of human umbilical-vein endothelial cells with the epithelial cell line A549 [21]. In that case the granulocytes were prelabelled with carboxyfluorescein diacetate succinimidyl diester (CFDA-SE), Molecular Probes, Eugene, OR, USA), to facilitate the counting of adherent granulocytes in randomly chosen visual fields by fluorescence microscopy (× 10/NA 0.3, objective Axiophot; Zeiss) as described [22].

### E-selectin expression analysis by FACS

glEND.2 cells were seeded on 48-well plates (4 × 10^4^) in complete low-glucose DMEM and grown to 80% confluence. ALP treatment was performed after a PBS washing step with 2 to 12 μM ALP for 2 hours at 37°C. Medium was removed and the cells were washed twice with PBS and activated for 4 hours with 20 ng/ml IL-1β. E-selectin expression was detected on staining with a rat anti-mouse CD62E-biotin (dilution 1:400; BD Biosciences Pharmingen, San Diego, CA, USA) antibody and phycoerythrin-labelled avidin (dilution 1:1,000; BD Biosciences Pharmingen). After a final centrifugation, the cells were resuspended in 1 ml of PBS and their fluorescence was measured by flow cytometry in a Coulter Epics XL flow cytometer (Beckman CoulterCorp., Miami, FL, USA). For the analysis, the gates of forward and sideways light scatter were set on the glEND.2 cell population. The average of E-selectin expression in the endothelial cell population was expressed as the mean of the fluorescence intensity.

### Analysis of nuclear NF-κB DNA binding activity

glEND.2 cells were grown to 80% confluence in 75 cm^3 ^culture flasks and treated with different concentrations of ALP (250 nM to 10 μM) for 2 hours at 37°C. After an intensive washing step, cells were activated with 10 ng/ml IL-1β for 1 hour. Subsequently, electrophoretic mobility-shift assay (EMSAs) of nuclear extracts from ALP-treated and untreated glEND.2 cells were prepared as described [23]. In brief, cells were resuspended in 200 μl of hypotonic lysis buffer A (10 mM HEPES pH 7.9, 10 mM KCl, 0.1 mM EDTA, 1 mM dithiothreitol) and incubated on ice for 15 minutes before the addition of 5 μl of 10% Nonidet P40. After vortex-mixing, the nuclei of the lysed cells were collected by centrifugation, washed once in buffer A and subjected to a subsequent protein extraction procedure by vortex-mixing vigorously in 50 μl of hypertonic extraction buffer B (10 mM HEPES pH 7.9, 400 mM NaCl, 1 mM EDTA, 1 mM dithiothreitol). The nuclear proteins were obtained by collecting the supernatants after centrifugation. The protein concentration of each extract was measured with the micro-bicinchoninic acid (BCA) assay reagents from Pierce (Rockford, IL, USA).

NF-κB binding reactions were performed with 5 μg of nuclear proteins and a ^32^P-labelled double-stranded oligonucleotide containing the NF-κB consensus sequence derived from the mouse κ intronic enhancer (5' -GATCAGAGGGGACTTTCCGAG-3'). Approximately 20,000 c.p.m. of the labelled double-stranded oligonucleotide was applied to each binding reaction. After incubation for 10 minutes at room temperature the nuclear proteins were separated by electrophoresis on a 5% nondenaturing polyacrylamide gel. For specificity, control blocking experiments were run with unlabelled specific double-stranded oligonucleotides or a respective mutant variant (5' -CATCAGAGGCGACTTTCCGAGGGATG-3') at 10-fold and 100-fold molar excess of the labelled NF-κB probe. Subsequently, the electrophoresis gels were dried, exposed on a PhosphoImager plate and analysed with a FLA-3000 laser-based imaging system (Fujifilm Europe GmbH, Düsseldorf, Germany). For normalization of the nuclear translocation of NF-κB in a quantitative analysis, aliquots (5 μg) of the nuclear proteins were assessed in parallel for their binding activity to a ^32^P-labelled double-stranded oligonucleotide containing the specific recognition sequence of the constitutively active transcription factor Oct-1 (5' -CTGTCGAATGCAAATCACTAGAAG-3'). The numerical proportion between NF-κB-binding and Oct-1-binding activity as determined by the PhosphoImager in c.p.m. served as the parameter of NF-κB activation.

### NF-κB-luciferase reporter assay

For transient transfection, 4× 10^4 ^glEND.2 cells grown in 24-well trays were transfected with a NF-κB reporter plasmid, pBIIx-luc [24,25], using the Fugene6 transfection reagent (Roche Diagnostics, Mannheim, Germany). All DNA/Fugene6 incubations were performed at a ratio of 1.5 μg of DNA to 3 μl of Fugene6 in accordance with the manufacturer's recommended protocol, in DMEM (PAA Laboratories, Pasching, Austria). After 24 hours, transfected and control glEND.2 cells were treated for 2 hours with 8 to 20 μM ALP. The activation of the luciferase reporter gene was induced by 10 ng/ml IL-1β for 4 hours and monitored by the application of a commercial luciferase reporter assay. On cell lysis, luciferase activity was determined in accordance with the instructions of the manufacturer of the luciferase assay kit (Promega, Mannheim, Germany) with a luminometer (Luminat LB 9501; Berthold). Protein concentrations of cell lysates were determined with the bicinchoninic acid micro-assay method (Pierce, Bonn, Germany).

## Results

### Effect of ALP on initial leukocyte-endothelial-cell interaction in the synovial microcirculation after anti-CII antibody transfer

**Figure 1:**
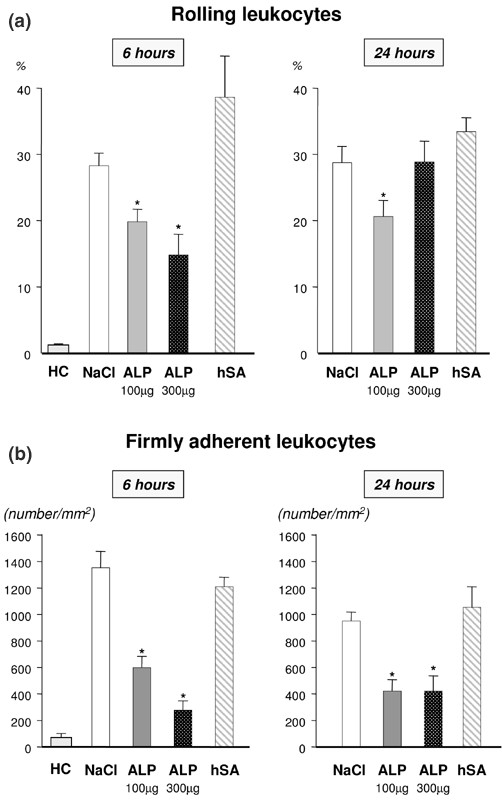
Inhibition of leukocyte interaction with the postcapillary endothelium by ALP *in vivo*. **(a) **Inhibition of leukocyte rolling along the postcapillary endothelium by antileukoproteinase *in vivo*. The results show the fraction of leukocytes rolling along the endothelium of postcapillary synovial venules (expressed as a percentage of all passing leukocytes) in anti-collagen II (CII) antibody-induced DBA1/J mice pretreated with either antileukoproteinase (ALP; 100 and 300 μg), human serum albumin (hSA, 300 μg) or NaCl in comparison with healthy mice (HC) that did not receive the antibody challenge. Intravital fluorescence microscopy of the knee joints was performed at 6 and 24 hours after the systemic transfer of anti-CII mAb. Results are shown as means and SEM for *n *= 8 animals per group; *p < 0.05. **(b) **Inhibition of leukocyte adherence to the postcapillary endothelium by ALP *in vivo*. The results show the number of leukocytes adherent to the endothelium of postcapillary synovial venules (expressed as cells per mm^2 ^endothelial surface) in anti-CII antibody-induced DBA1/J mice pretreated with either ALP (100 and 300 μg), hSA (300 μg) or NaCl in comparison with healthy mice (HC) that did not receive the antibody challenge. Intravital fluorescence microscopy of the knee joints was performed at 6 and 24 hours after the systemic transfer of anti-CII mAb. Results are shown as means and SEM for *n *= 8 animals per group; *p < 0.05.

**Figure 2:**
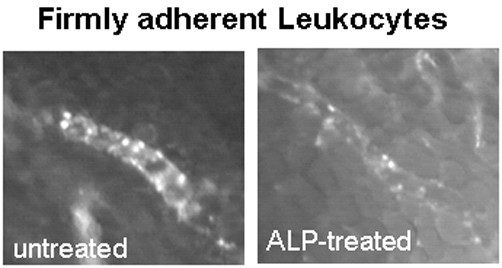
Imaging of adherent leukocytes *in vivo*. The figure shows a representative fluorescence microscope image (original magnification × 306) of a postcapillary venule showing an increased number of cells adhering to the wall of the postcapillary venule in a control animal that received the anti-collagen II (CII) antibodies and a pre-treatment with NaCl compared to an antileukoproteinase-treated mouse at 24 hours after anti-CII mAb transfer.

Although the systemic administration of the anti-CII mAb did not cause clinical signs of arthritic disease in the knee joints investigated by intravital microscopy within the first 24 hours, inflammatory cell responses in the synovial tissue were clearly provoked. Activation by inflammatory stimuli after antibody transfer to the mice was indicated by increases in leukocyte rolling along and attachment to the venular endothelium (Figure 1). These microcirculatory alterations are not spontaneously occurring phenomena in the knee joints of uninduced control mice (Figure 1a) [17]. However, the enhanced leukocyte-endothelial-cell interaction induced by the transfer of anti-CII mAb was significantly (*p *< 0.05) suppressed in the ALP-pretreated mice. This blocking effect of ALP on leukocyte adherence to the vessel intima (Figure 2) occurred on both the initial rolling and the subsequent phase of firm adhesion. Dose dependence of inhibition was clearly detectable at 6 hours after the transfer of anti-CII mAb. Later in the observation period the suppressive effect declined. However, in this respect the ALP blockade of firm adhesion was more pronounced and persisted for the entire observation period of 24 hours (Figure 1). The blocking effect was ALP-specific because human serum albumin (hSA) as an irrelevant control protein affected neither the antibody-induced increase in leukocyte rolling nor the firm leukocyte adhesion. This result is consistent with an inhibitory effect of ALP on both selectin-dependent and integrin-dependent interactions of leukocytes with the activated synovial endothelium. With regard to the observed suppression of firm leukocyte adhesion, the effect *in vivo *complies very well with the earlier described ALP-induced blockade of leukocyte β_2_-integrin activation. However, the impairment of leukocyte rolling by ALP was rather surprising and required further investigation.

### Effect of ALP on leukocyte adherence to cytokine-activated endothelial cells *in vitro*

**Figure 3:**
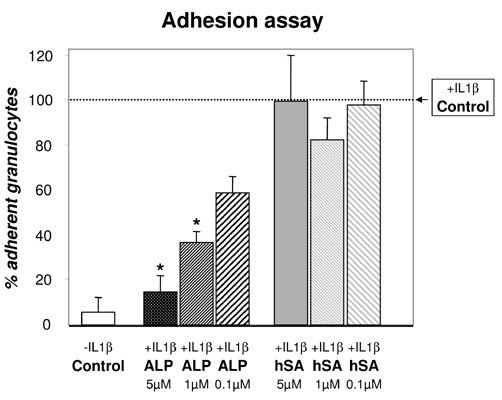
Inhibition of leukocyte attachment to IL-1β-activated glEND.2 cells by antileukoproteinase *in vitro*. The adherence of human granulocytes to layers of glEND.2 cells was assessed microscopically. The quantitative measure for leukocytes adhering to the glEND.2 cells under the respective incubation conditions is expressed as a percentage of the positive control, which is the mean number of adherent cells to IL-1β-activated endothelium in medium (+IL-1β control; stimulation with 20 ng/ml IL-1β for 4 hours). The percentages of adherent leukocytes to glEND.2 cells were derived from eight independent experiments and are expressed as means and SEM for the following experimental conditions: no stimulus (- IL-1β control), IL-1β stimulation after pretreatment with either antileukoproteinase (ALP) or with human serum albumin (hSA) (0.1, 1 or 5 μM) as a control protein for 2 hours. The inhibitory effect of pretreatment with ALP on granulocyte adherence was statistically significant at 1 and 5 μM (*p < 0.01; analysis of variance with correction for multiple testing).

The model of the IL-1β-activated murine endothelial cell line glEND.2 permitted the analysis of effects of ALP on granulocyte attachment under conditions that had earlier been proved to depend partly on selectin-mediated interactions [19]. Preincubation of the endothelial layer with ALP (in 20 μl of PBS carrier solution dissolved in DMEM to a final volume of 200 μl) instead of medium control (200 μl DMEM) before IL-1β activation resulted in a significantly decreased attachment of purified human granulocytes (the total cell number per chamber was 2 × 10^5 ^per 200 μl) in eight independent experiments, as shown in Figure 3. In Figure 3 the quantitative measure for leukocytes that adhered to the glEND.2 cells under the respective incubation conditions are expressed as percentages of the positive control (3,782 ± 624 cells in four randomly chosen visual fields), which is the mean number of cells adhering to IL-1β-activated endothelium in medium (DMEM). Figure 3 clearly shows the highly significant decrease (*p *< 0.01; ANOVA with Dunnett's correction for multiple testing) in leukocytes adhering to IL-1β-activated glEND.2 cells under pretreatment conditions with ALP (1 μM, 36.25 ± 6.67% (mean ± SEM)) compared with those with the respective control protein hSA (5 μm, 99.66 ± 18.88%; unstimulated glEND.2 cell control, 10.99 ± 5.79%).

**Figure 4:**
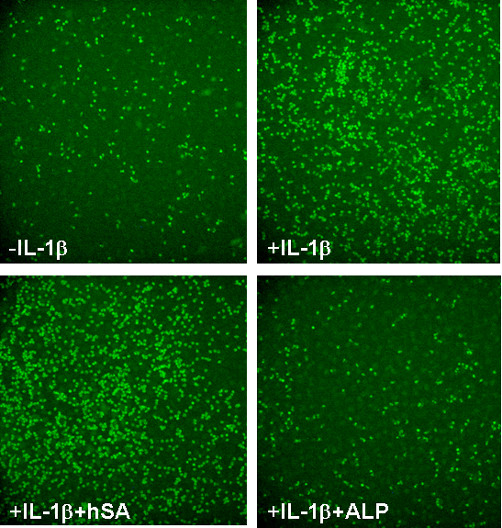
Inhibition of leukocyte attachment to IL-1β-treated EA.hy 926 cells by antileukoproteinase *in vitro*. Adherence of human CFDA-SE (carboxyfluorescein diacetate succinimidyl diester)-labelled granulocytes to layers of EA.hy 926 cells was assessed by fluorescence microscopy. A comparison of granulocyte adherence to EA.hy 926 cells that were either untreated (- IL-1β) or cytokine-stimulated (+IL-1β) is shown. Pretreatment of EA.hy 926 cells with 5 μM antileukoproteinase (ALP) (+IL-1β +ALP) but not with 5 μM hSA (+IL-1β +hSA) resulted in a marked decrease in granulocyte adhesion.

These results suggest that pretreatment with ALP has a blocking effect on the subsequent cytokine-induced activation of selectin-dependent endothelial interactions with leukocytes. Moreover, the inhibitory effect of ALP is likely to affect primarily the endothelial cell because a thorough washing procedure preceded the addition of the isolated granulocytes, to ensure that the cellular interaction took place after the removal of the cytokine and the ALP from the medium. Additional experiments (*n *= 8) performed with human CFDA-SE-labelled granulocytes and a human endothelial cell line EA.hy 926, an accepted *in vitro *model system for the investigation of leukocyte-endothelial-cell interaction [21,22], confirmed the blocking effect of ALP on granulocyte adhesion to the IL-1β-activated endothelial cells. The quantitative assessment of the experiments, from which four representative panels are shown in Figure 4, clearly revealed the significant decrease (*p *< 0.01; ANOVA) in adherent leukocytes to IL-1β-activated EA.hy 926 cells pretreated with ALP (5 μM, 32.1 ± 5.8%) compared with hSA (5 μM) control (97.2 ± 15.2%).

### Effect of ALP on IL-1β-induced E-selectin upregulation on endothelial cells

**Figure 5:**
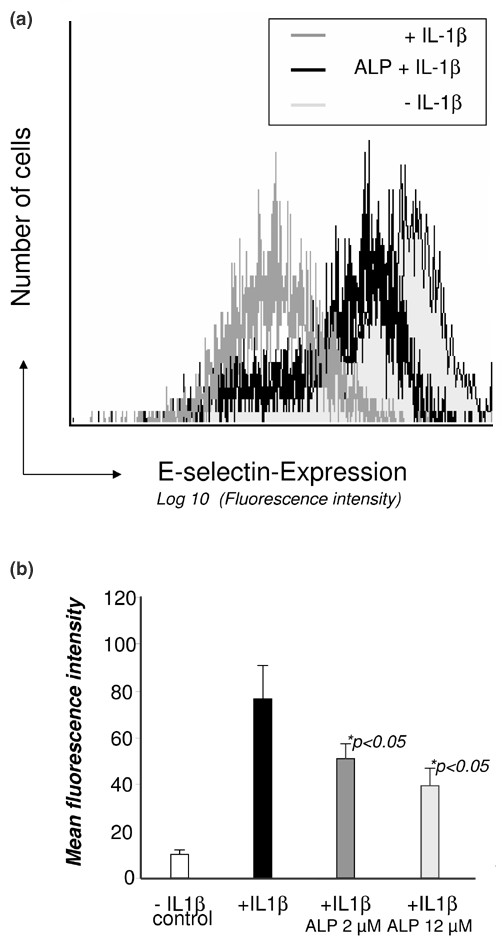
Inhibition of IL-1β-induced upregulation of E-selectin in glEND.2 cells by ALP. **(a) **Inhibition of IL-1β-induced upregulation of E-selectin in glEND.2 cells by antileukoproteinase (ALP). Determination of E-selectin expression on glEND.2 cells was performed by FACS analysis after staining with a biotinylated anti-E-selectin mAb and phycoerythrin-labelled-streptavidin. The histograms obtained after gating on glEND.2 cells for forward and sideways light-scattering properties and counting of 5,000 events demonstrate the increasing surface expression of E-selectin in response to stimulation with IL-1β (curve shifted furthest to the right) compared with the unstimulated control (leftmost curve). The intermediate curve represents the E-selectin distribution on glEND.2 cells pretreated with ALP (2 μM) before stimulation with IL-1β. **(b) **Inhibition of E-selectin expression by ALP. Mean fluorescence intensities were determined as a measure of the E-selectin expression levels in six independent experiments and used to calculate the mean and SEM for the following experimental conditions of glEND.2 cell activation: no stimulus (- IL-1β control), IL-1β stimulus (+IL-1β), IL-1β stimulation after pretreatment with ALP for 2 hours (+IL-1β ALP 2 μM and +IL-1β ALP 12 μM). The inhibitory effect of pretreatment with ALP on E-selectin expression was statistically significant for both concentrations of ALP (*p < 0.05).

On the basis of the above experimental evidence for the capacity of ALP to block leukocyte interaction with cytokine-activated endothelium to the same extent as specific E-selectin mAbs under similar conditions [19], we considered the possibility of a direct E-selectin-modulation by ALP and investigated it further. Thus, E-selectin expression on glEND.2 cells that were either preincubated with medium or ALP before stimulation for 4 hours with IL-1β was assessed in parallel by FACS analysis of the cells stained with anti-E-selectin. The fluorescence intensities were determined in six independent experiments; an example is shown in Figure 5a. The results of the statistical analysis of the mean fluorescence intensities are shown. In Figure 5b we demonstrate a significant decrease in the cell-surface expression of E-selectin on glEND.2 cells that had been exposed to ALP before the cytokine stimulus (2 μM ALP, 52.2 ± 4.7 (mean ± SEM); 12 μM ALP, 39.6 ± 6.5) compared with the respective controls without ALP pretreatment (76.7 ± 9.7; control of unstimulated glEND.2 cells, 10.2 ± 0.7). Our investigations therefore support a direct suppression of IL-1β-induced E-selectin upregulation in glEND.2 cells by ALP.

### Effect of ALP on IL-1β-induced NF-κB activation in endothelial cells

**Figure 6:**
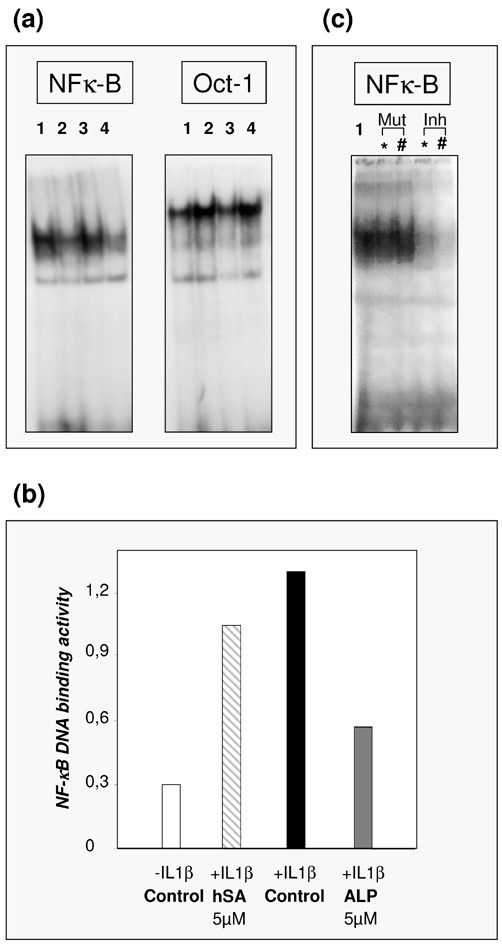
Inhibition of IL-1β-induced NF-κB activation in glEND.2 cells by antileukoproteinase. **(a) **The left panel shows an electrophoretic mobility-shift assay (EMSA) performed with nuclear extracts from unstimulated control cells (lane 4) and IL-1β-stimulated glEND.2 cells that had been preincubated in either the absence (lane 1) or the presence of antileukoproteinase (ALP; lane 2) or human serum albumin (hSA; lane 3) as an irrelevant control protein. Pretreatment of the glEND.2 cells with ALP was associated with a clearly decreased nuclear translocation of NF-κB reflected by the weakened intensity of the corresponding band shifts of the radiolabelled NF-κB-specific oligonucleotides. For comparison a concomitantly developed EMSA from the same experiment shows the electrophoretic mobility of the constitutively active transcription factor Oct-1 (right panel; lanes loaded as described for the left panel). **(b) **The densitometric evaluation of an independently performed EMSA experiment depicts the difference in NF-κB signal intensities between unstimulated control (- IL-1β control) and IL-1β-stimulated glEND.2 cells preincubated with either 5 μM ALP or 5 μM hSA. As a quantitative measure of the nuclear translocation of NFκ-B the figure shows the relative DNA-binding activity of NF-κB. This parameter represents the numerical proportion between NF-κB-binding and Oct-1-binding activity in c.p.m. The protein binding to the radiolabelled specific probe for the constitutively active transcription factor Oct-1 served as an internal standard for normalization of the nuclear extracts. **(c) **An additional specificity control for the NF-κB EMSA. Before electrophoretic separation, nuclear extracts from IL-1β-stimulated glEND.2 cells were incubated not only with the radiolabelled NF-κB but also with two different concentrations of unlabelled competitor probes. The results of the blocking experiments of the NF-κB EMSA with an unlabelled NF-κB-probe (Inh) (showing blocking at 10-fold (*) and 100-fold (^#^) molar excess) and a mutant NF-κB probe (Mut) that did not suppress the NF-κB signal at 10-fold and 100-fold molar excess, respectively, are shown. Lane 1 was loaded with a sample without competing probe as in (a).

NF-κB has a pivotal role in the cytokine-induced signalling pathways that lead to an upregulation of E-selectin on the cell membrane [12]. We therefore analysed the possibility that ALP interfered with IL-1β-induced NF-κB-signalling in glEND.2 cells. EMSAs were performed with nuclear extracts from unstimulated control cells and IL-1β-stimulated glEND.2 cells that had been preincubated in either the presence or the absence of ALP. As shown in Figure 6a, pretreatment of the glEND.2 cells with ALP, but not with the irrelevant control protein hSA, was associated with a markedly decreased nuclear NF-κB DNA-binding activity. As a control, a parallel EMSA from the same experiment showing the electrophoretic mobility of Oct-1 binding nuclear proteins was performed. The control EMSA exhibited neither an IL-1β-induced increase in Oct-1 nuclear binding activity nor a suppressive effect on the dependence on pretreatment of the cells with ALP or hSA. The densitometric evaluation of another independently performed EMSA experiment also revealed a clear difference in NF-κB signal intensities between IL-1β-stimulated glEND.2 cells preincubated with either ALP (5 μM) or hSA (5 μM) (Figure 6b).

**Figure 7:**
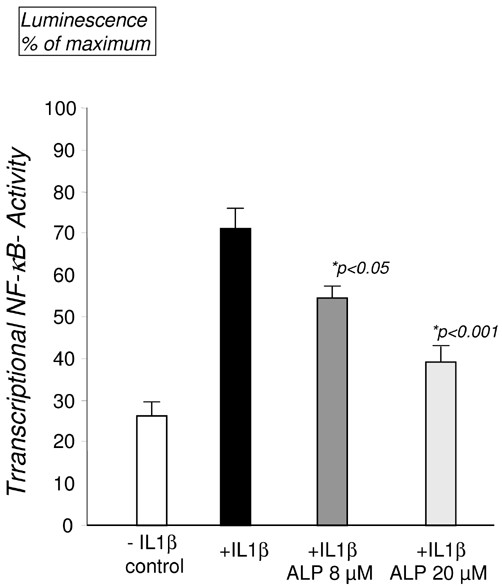
Inhibition of IL-1β-induced NF-κB transcriptional activity in glEND.2 cells by antileukoproteinase. Effects of antileukoproteinase (ALP) on glEND.2 cells that had been transfected with an NF-κB-luciferase reporter gene construct were analysed separately. The determination of luminescence signals in the luciferase assay of the lysed cells permitted the quantification of the IL-1β-induced upregulation of transcriptional NF-êB-activity (expressed as a percentage of luminescence maximum). The stimulatory cytokine effect on NF-κB activation (+IL-1β) above the level of the unstimulated control (- IL-1β control) was significantly suppressed by ALP (*p < 0.05) in a concentration-dependent manner (+IL-1β ALP 8 μM and +IL-1β ALP 20 μM). Results are means and SEM for 16 independent experiments.

We also controlled for the specificity of the NF-κB EMSA by competition experiments containing the radiolabelled NF-κB probe and unlabelled probes. The results of the blocking experiments of the NF-κB EMSA with an unlabelled NF-κB probe (showing blocking at 10-fold and 100-fold molar excess) and a mutant NF-κB probe that did not suppress the NF-κB signal at 10-fold and 100-fold molar excess, respectively, are shown in Figure 6c, providing clear evidence for the specificity of the NF-κB EMSA. To further investigate the influence of ALP on the transcriptional activity of NF-κB, endothelial glEND.2 cells were transfected with an NF-κB-dependent luciferase reporter gene and the luciferase activity in lysed cells was quantified. As expected, the transcriptional activity of NF-κB was strongly induced by stimulation with IL-1β. Preincubation with ALP dose-dependently inhibited the IL-1β-induced transcriptional NF-κB activity, as shown in Figure 7. This result is in agreement with the finding of decreased nuclear NF-κB DNA-binding activity after treatment with ALP.

## Discussion

ALP has previously been shown to exert a variety of anti-inflammatory effects beyond its function as an inhibitor of granulocytic serine proteinases [3-5,13]. We have recently demonstrated its antiarthritic effect in the CAIA model as well as a suppressive effect on immune-complex-induced activation of β_2_-integrins in leukocytes. Because of the modulatory potential of ALP on the function of integrins that are crucially involved in leukocyte extravasation [5], the present study focused on its interference with leukocyte-endothelial-cell interactions. Accordingly, a blocking effect of ALP on the firm leukocyte adhesion induced by anti-CII mAb transfer *in vivo *was detectable. This effect is consistent with the well established inhibitory effect of ALP on the cytoskeletal reorganization of stimulated granulocytes and the accompanying suppression of conformational changes of the β_2 _chain of the integrins LFA-1 (CD11a/CD18) and Mac-1 (CD11b/CD18) [5], because the activation of these molecules is a requirement for firm leukocyte attachment to the activated endothelial lining [7-9]. Interestingly, a slight but significant decrease in firm leukocyte adhesion to the vessel wall is also detectable in the NaCl-control cohort during the interval between 6 and 24 hours after anti-CII antibody transfer (from 1,354 ± 125 (mean ± SEM) to 953 ± 67). However, this tendency did not continue spontaneously in the later course. Measurements of leukocyte attachment at 72 hours after antibody transfer (928 ± 84) suggest that the level reached at 24 hours after an initial decline from the early peak already represents the plateau value that is highly elevated above normal and maintained until at least 72 hours, a time point coinciding with first clinical signs of arthritis in the arthritis model induced by anti-CII antibody.

In addition to the suppression of firm leukocyte adhesion in ALP-pretreated mice, inhibitory effects on the rolling phase of the leukocytes along the vessel wall were observed by intravital microscopy. During an inflammatory response, leukocytes roll along the endothelial layer of cytokine-activated venules before they arrest, adhere and transmigrate. CD18 integrins, as known targets of ALP action [5], have been shown to be unable to mediate leukocyte rolling independently of selectins [26,27]. In this respect, selectins seem to ensure an appropriate decrease in rolling velocity that enables the leukocytes to spend a critical time span in close contact with the endothelium in most tissues [26]. This decelerated rolling allows proinflammatory cytokines to be displayed by the endothelium, for example by means of proteoglycans [28], to engage their respective receptors on the leukocyte membrane, thereby leading to the activation of β_2_-integrins. The subsequent β_2_-integrin-dependent interactions with endothelial ligands (such as ICAM-1) stabilize the retardation of rolling, provoke arrest and cause firm adhesion as preconditions for the final transmigration through the blood vessel wall [29].

In accordance with this complex interwoven action of selectins and CD18 integrins in leukocyte rolling and adhesion, it was consistent to propose blocking effects of ALP on leukocyte rolling *in vivo *as an additional interference with selectin interactions beyond the already known suppression of leukocyte integrin activation [5]. In an *in vitro *assay of E-selectin-dependent leukocyte attachment to the IL-1β-activated glEND.2 cells, the significantly decreased adherence to the ALP-pretreated endothelial layer provides first evidence for ALP-induced modulation of the endothelial function of E-selectin. Moreover, our investigations have elucidated a suppression of the cytokine-induced upregulation of E-selectin on the endothelial membrane by ALP. E-selectin expression is regulated primarily at the level of transcriptional activation, a process mediated by a series of tandem NF-κB sites in the promoter of this gene [30]. Stimuli such as IL-1β can rapidly enhance the expression of the E-selectin gene in endothelial cells by inducing a cytoplasmic-nuclear shuttling of NF-κB that causes transcriptional activation on binding to the E-selectin promoter [12,31].

Although suppressive effects of ALP on the NF-κB signalling pathway by inhibitory mechanisms that are as yet unresolved had already been described in other experimental systems *in vivo *and *in vitro *[6,13], it was not at all clear that this action also had the potential to counteract IL-β-induced endothelial cell activation. Therefore this newly uncovered interference with the IL-β-induced nuclear translocation of transcriptionally active NF-κB is likely to account for the observed suppression of cytokine-activated E-selectin upregulation by ALP. However, the precise molecular mechanism underlying the suppressive effect of ALP on IL-1β-induced NF-κB activation in endothelial cells remains enigmatic. Similarly, the suppressive effect of ALP on lipopolysaccharide-induced NF-κB activation in monocytic cells (U937) has been shown to imply an inhibition of the cytoplasmic degradation of IκBα. However, ALP did not suppress the phosphorylation or ubiquitination of IκBα and did not exhibit activities of a broad-spectrum inhibitor of the proteasomal degradation machinery [32].

More recently, the cytoplasmic localization of ALP after rapid uptake from the extracellular space and its subsequent translocation into the nucleus, associated with direct binding to nuclear NF-κB sites, have been described in U937 cells [33]. However, we could not detect in our EMSA shifts (Figure 6a), performed with NF-κB-specific probes, any ALP-oligonucleotide complexes in the expected lower molecular mass range, rendering the possibility of direct nuclear binding of ALP to NF-κB sites and thus competition with p65 interaction a rather unlikely explanation for our results obtained in endothelial cells. Whether ALP induces other IκB proteins or exerts its suppressive effect on NF-κB through the activation of alternative signalling pathways remain interesting possibilities. Irrespective of the precise mode of ALP action, NF-κB inhibition and its closely associated suppression of cytokine-activated E-selectin upregulation provide an attractive explanation for the observed inhibitory effects on selectin-dependent leukocyte attachment to murine glEND.2 and human EA.hy 926 cells *in vitro*. Moreover, it is rather likely that modulation of selectins is also involved in decreased leukocyte rolling after anti-CII mAb transfer in ALP-treated mice in comparison with the control *in vivo*. In addition, the suppression by ALP of IL-1β-induced NF-κB signalling is likely to have a much broader spectrum of anti-inflammatory effects on the endothelial cells than the downregulation of E-selectin expression investigated as an example because the inhibited signalling pathway is critical for the upregulation of a plethora of adhesion molecules and inflammatory mediators [34].

This broad suppressive effect on the activation of the endothelial layer cooperates with already known properties of ALP to downmodulate β_2_-integrin-dependent proinflammatory functions in leukocytes [5] to counteract leukocyte extravasation. Indeed, this inhibitory effect of ALP on leukocytic tissue infiltration has already been demonstrated in a variety of models such as streptococcal cell wall arthritis [13], CAIA [5], reverse passive cutaneous Arthus reaction (B. Sehnert, A. Cavcic, and H. Burkhardt, unpublished results) and inflammatory organ injury in a murine ischaemia/reperfusion model [14]. This potential of ALP to inhibit leukocyte extravasation in response to quite diverse inflammatory stimuli suggests its extended role as a regulatory molecule of innate immunity. Of course, interesting questions remain to be answered in further studies: the effect of ALP on the regulation of anti-inflammatory cytokines such as TGF-β, on vascular permeability or its potential to interfere with trafficking of other cells of the innate and adaptive immune system such as lymphocytes, monocytes, dendritic cells and mast cells. The answers to these questions will help in judging therapeutic potential in future treatment strategies. However, the effect of ALP on both β_2_-integrins on leukocytes and endothelial cell adhesion molecules such as E-selectin suggests a modulatory role of cell-cell adhesion mechanisms without complete suppression, because otherwise the combination of blocking selectins and integrins would result in a complete lack of polymorphonuclear cells adhesion. ALP therefore offers an attractive possibility for modulating the immune response. In this respect our work has uncovered for the first time the suppressive effects of ALP on selectin-dependent and integrin-dependent leukocyte adhesion events to the vessel walls that cooperate in the prevention of subsequent leukodiapedesis.

## Conclusion

The newly uncovered ability of ALP to interfere with cytokine signalling and with the upregulation of adhesion molecules in endothelial cells probably contribute to its protective effects on immune-complex-mediated tissue injury. These new insights into the spectrum of anti-inflammatory actions of ALP might prove useful in the development of therapeutic interventions in endothelial barrier dysfunction caused by misdirected leukocyte activation in the joints or at extra-articular sites of immune-complex deposition in systemic disease.

## Abbreviations

ALP = antileukoproteinase; ANOVA = analysis of variance; CAIA = anti-collagen II antibody-induced arthritis; CFDA-SE = carboxyfluorescein diacetate succinimidyl diester; CII = collagen type II; DMEM = Dulbecco's modified Eagle's medium; EMSA = electrophoretic mobility-shift assay; FACS = fluorescence-activated cell sorting; hSA = human serum albumin; IL = interleukin; mAb = monoclonal antibody; NF = nuclear factor; PBS = phosphate-buffered saline.

## Competing interests

The authors declare that they have no competing interests.

## Authors' contributions

BS performed the *in vitro *assays (cell adhesion assays, FACS analysis, reporter gene assays) and the statistical analysis. PG and AK performed the animal experiments and intravital fluorescence microscopic analysis. RV conducted the electromobility shift experiments and contributed to the design of the reporter gene assays. KSN produced and purified the CII-specific mAbs for the transfer experiments. SI, BV and RHo participated in the design and coordination of the animal experiments and contributed to the preparation of the manuscript. RHa established the endothelial cell line and participated in the design of the adhesion assays. HB conceived the entire study, participated in the design, coordination and analysis of the *in vitro *studies and drafted the manuscript. All authors read and approved the final manuscript.
